# Prognostic Value of 48-h Biomarker Changes in Sepsis Mortality

**DOI:** 10.3390/jcm14248651

**Published:** 2025-12-06

**Authors:** Yeliz Özdemir, Özkan Özmuk, Şebnem Çalık, Selma Tosun

**Affiliations:** 1 Department of Infectious Diseases and Clinical Microbiology, Izmir City Hospital, Izmir 35540, Türkiye; 2 Department of Intensive Care Unit, Izmir City Hospital, Izmir 35540, Türkiye; 3 Department of Infectious Diseases and Clinical Microbiology, University of Health Sciences, Izmir Bozyaka Training and Research Hospital, Izmir 35170, Türkiye

**Keywords:** sepsis, intensive care units, biomarkers, prognosis, mortality

## Abstract

**Background:** Sepsis remains a major cause of morbidity and mortality in intensive care units (ICUs). Although various scoring systems and biomarkers have been studied, the prognostic significance of early dynamic changes in laboratory parameters remains unclear. This study aimed to investigate the prognostic value of 48 h changes in routinely monitored biomarkers for in-hospital mortality in septic patients. **Methods:** This retrospective, single-center study was conducted in the Anesthesiology and Reanimation ICU of a tertiary teaching hospital. A total of 174 adult patients (≥18 years) diagnosed with sepsis according to SEPSIS-3 criteria between January 2017 and December 2022 were included. Laboratory data were recorded at ICU admission and after 48 h. Patients who died within 48 h or had missing follow-up data were excluded. Receiver operating characteristic (ROC) analysis and logistic regression models were used to assess the prognostic performance of clinical and laboratory parameters. **Results:** The median age was 71 years, and 58% of patients were male. Comorbidities were present in 76% patients, and malignancy was associated with higher mortality (*p* = 0.012). The overall in-hospital mortality rate was 58.6%. Inappropriate empirical antibiotic therapy significantly increased mortality risk (*p* = 0.001). Non-survivors had higher baseline SOFA and APACHE II scores. At 48 h, mortality was associated with increased procalcitonin, lactate, and CRP/albumin ratio and greater albumin decline. ROC analysis identified procalcitonin ≤ 28% decrease, lactate > 23% increase, albumin > 7% decrease, and CRP/albumin ratio > 31% increase as optimal cutoffs. Multivariate analysis revealed SOFA score > 6, inappropriate antibiotic therapy, procalcitonin ≤ 28% decrease, lactate > 23% increase, and platelet > 37% decrease as independent mortality predictors. The change in albumin level was included in the model but was not statistically significant. **Conclusions:** Forty-eight–hour biomarker changes, particularly in lactate and platelet count, may provide complementary prognostic information to baseline SOFA scores and may support early risk stratification in sepsis. These findings should be considered exploratory and require confirmation in prospective multicenter studies before clinical implementation.

## 1. Introduction

Sepsis is a clinical syndrome characterized by complex pathophysiological processes, driven by systemic inflammation and concurrently affecting multiple organ systems [1]. Despite advances in intensive care practices and antimicrobial treatments, sepsis remains a significant health problem worldwide, with high mortality and morbidity rates. Mortality rates associated with sepsis in intensive care units (ICUs) range from 20% to 50%, and can rise as high as 67% in some critical patient groups [2,3,4,5].

Prognostic scoring systems such as the Sequential Organ Failure Assessment (SOFA) and the Acute Physiology and Chronic Health Evaluation II (APACHE II) are widely used to assess disease severity and estimate prognosis. However, their predictive accuracy can be limited in heterogeneous patient populations, as these scores represent static assessments that may not fully capture the dynamic evolution of sepsis [6,7]. In addition to these limitations, recent institutional quality-improvement initiatives—such as the Princess Sepsis Code (PSC), developed at La Princesa University Hospital and grounded in Pronovost’s cultural transformation model—have shown that structured, system-level programs can enhance early sepsis recognition and improve adherence to care bundles, ultimately reducing mortality. This shift underscores the increasing emphasis on dynamic, time-sensitive assessment in modern sepsis management [8].

In this context, biomarkers have become increasingly important for understanding the pathogenesis of sepsis, as well as for supporting clinical diagnosis and prognostic assessment. Recent studies have shown rising interest in markers that mirror the host response and the course of the disease. Procalcitonin (PCT), lactate, C-reactive protein (CRP), and platelet count are among the key biomarkers frequently used in the diagnostic and prognostic evaluation of sepsis [9]. PCT, which rises significantly during bacterial infections, reflects the severity of the systemic inflammatory response. Under normal conditions, PCT is produced by thyroid C cells; however, in severe infections such as sepsis, it is also secreted by monocytes and various parenchymal tissues [10]. Persistently elevated lactate levels, an indicator of tissue hypoperfusion and cellular oxygen deprivation, have been associated with poor prognosis in sepsis [11]. Thrombocytopenia, frequently observed during the course of sepsis, results from coagulopathy, immune dysregulation, and endothelial damage and is strongly correlated with increased mortality [12]. CRP, produced by hepatocytes during the systemic inflammatory response, is commonly used as a biomarker to monitor the progression of sepsis [13]. Moreover, composite indices such as the CRP-to-albumin ratio have been shown to provide additional prognostic value in predicting mortality and disease severity [14].

Throughout the dynamic course of sepsis, the host response evolves over time, with immunological and biological reactions varying across different stages of the disease. Consequently, the clinical value of prognostic biomarkers may also fluctuate during the course of illness [15]. Notably, changes in procalcitonin, CRP, and lactate levels within the first 24–48 h have been shown to possess stronger prognostic significance compared with their initial values. Therefore, not only baseline levels but also early dynamic changes in these biomarkers may offer valuable prognostic insights into mortality risk. Recently, Varga et al. (2024) highlighted that evaluating dynamic biomarker trajectories rather than single-time measurements enhances the accuracy of mortality prediction and complements clinical scoring systems in sepsis [16].

Although several prior studies have evaluated early changes in individual biomarkers such as PCT, lactate, albumin, or platelet count, these investigations have largely examined each marker separately or in limited combinations. However, the inflammatory, hemodynamic, and hematologic pathways activated during early sepsis progress simultaneously rather than independently. Thus, evaluating multiple biomarker trajectories within a unified analytical framework may provide a more holistic representation of early host response than single-marker assessments.

Building upon this evidence, the present study contributes to the existing literature by assessing the 48 h percentage change (Δ) in routinely monitored biomarkers (PCT, lactate, albumin, platelet count, and CRP/albumin ratio) within a real-world ICU cohort, and by exploring how these early trajectories relate to in-hospital mortality when considered alongside baseline SOFA scores and the appropriateness of empirical antibiotic therapy.

## 2. Materials and Methods

### 2.1. Study Design

A retrospective, single-center study was conducted in the Anesthesiology and Reanimation ICU of a tertiary hospital, including 174 adult patients (aged ≥ 18 years) diagnosed with sepsis according to the Sepsis-3 criteria and admitted between January 2017 and December 2022. Sepsis was defined as a suspected or confirmed infection accompanied by an acute increase of ≥2 points in the SOFA score.

Patients younger than 18 years, those who died within 48 h, cases with incomplete data, and postoperative or trauma patients were excluded. Patients who died within the first 48 h were excluded because serial (baseline and 48 h) biomarker measurements were required to assess dynamic changes. All consecutive eligible patients meeting the inclusion criteria during the study period were analyzed to minimize potential selection bias.

The exclusion of early deaths was based solely on methodological feasibility rather than clinical outcome, ensuring the integrity of the cohort.

To evaluate the sensitivity and robustness of the multivariable analysis performed in the 174-patient cohort, additional models were constructed by incorporating the 14 patients who died within the first 48 h, allowing assessment of how survivorship bias might influence predictor stability and model performance.

Ethical approval was obtained from the Clinical Research Ethics Committee of Health Sciences University, Izmir Bozyaka Training and Research Hospital (Decision No: 2022/166). The study was conducted in accordance with the Declaration of Helsinki, and the requirement for informed consent was waived owing to its retrospective design.

### 2.2. Data Collection

Patient characteristics, including age, sex, comorbid conditions, and initial SOFA and APACHE II scores, were documented. Baseline values were defined as clinical and laboratory data obtained within the first 6 h of ICU admission. As part of the laboratory evaluations, complete blood counts (leukocytes, neutrophils, lymphocytes, platelets), biochemical parameters (creatinine, bilirubin, albumin), and inflammatory biomarkers (PCT, CRP, lactate) routinely monitored in the ICU were analyzed. These biomarkers were evaluated at baseline and again 48 h later.

Relative changes between baseline and 48 h were calculated for each biomarker. Delta values for PCT, CRP, lactate, albumin, neutrophils, lymphocytes, platelets, neutrophil-to-lymphocyte ratio (NLR), platelet-to-lymphocyte ratio (PLR), and CRP-to-albumin ratio were defined as the percentage change calculated by subtracting the values at 48 h from the baseline values. Delta values were calculated as percentage change using the following formula: Δ (%) = [(value at 48 h − baseline value)/baseline value] × 100.

A positive Δ value (+) indicates an increase from baseline to 48 h, whereas a negative Δ value (−) indicates a decrease. For clarity, the direction of change was also denoted using arrows in tables: ↑ for increase and ↓ for decrease.

All patients underwent daily evaluations during ICU rounds, jointly performed by an infectious diseases consultant, an infection control nurse, and an intensive care physician. Antimicrobial management was revised according to this multidisciplinary evaluation and aligned with contemporary guideline recommendations, while treatment appropriateness was assessed on the basis of these criteria [17,18,19]. Inappropriate empirical antibiotic therapy was defined as initial treatment not providing adequate coverage for the identified pathogen or not consistent with guideline-based empiric recommendations at the time of administration.

### 2.3. Statistical Analysis

Data were analyzed using SPSS (Statistical Package for the Social Sciences) Statistics version 26.0 (IBM Corp., Armonk, NY, USA). The normality of continuous variables was assessed using graphical methods, the Kolmogorov–Smirnov test, and sample size considerations. As the assumption of normal distribution was not met in all subgroups, comparisons between independent groups were performed using the Mann–Whitney U test. Continuous variables were reported as medians with interquartile ranges (IQR, 25–75%).

Receiver operating characteristic (ROC) curve analysis was conducted to determine the optimal cutoff values, which were identified according to the Youden index. These cutoffs were then used to generate dichotomous variables. Categorical variables were summarized as frequencies and percentages, and their distributions were compared using the Chi-square test or Fisher’s exact test, while univariate odds ratios (ORs) were calculated.

In the logistic regression analysis, six potential prognostic variables known in the literature to be associated with mortality were selected: SOFA score, 48 h change in procalcitonin, 48 h change in platelet count, 48 h change in lactate, 48 h change in albumin, and appropriateness of antibiotic therapy. All variables were dichotomized, and cutoff points were based on clinically meaningful thresholds. Using the enter method, all variables were included in the model simultaneously, thereby avoiding statistical bias associated with data-driven variable selection. To minimize the instability that commonly arises from stepwise or univariate-significance-based approaches, this limited predictor set was prespecified a priori on the basis of biological plausibility and prior evidence rather than selected through automated statistical algorithms. After evaluating the primary model performance, the analyses were supplemented with sensitivity analyses designed to quantify the impact of survivorship bias—one of the major methodological concerns of the study.

### 2.4. Sensitivity Analyses for Survivorship Bias

To quantitatively assess the impact of survivorship bias—one of the principal limitations of the study—comprehensive sensitivity analyses were designed:

1. Primary Model (Baseline Scenario):

Only patients who completed the 48 h follow-up and had available biomarker change data (*n* = 174) were included. This represents the original analysis.

2. Low-Risk Scenario:

Fourteen patients who died within the first 48 h were added to the analysis. Missing 48 h biomarker change values for these patients were imputed as belonging to the “good prognosis” or “low-risk” category.

For example, these patients were assigned:

- Procalcitonin change > 28% (favorable);
- Platelet change ≤ 37% (favorable);
- Lactate change ≤ 23% (favorable);
- Albumin change > 7% (favorable).

This reflects the assumption that early non-survivors would have had the best-possible biomarker trajectories.

3. High-Risk Scenario:

The same 14 early non-survivors were added, but missing 48 h biomarker change values were imputed as “poor prognosis” or “high-risk.”

Accordingly, these patients were assigned:

- Procalcitonin change ≤ 28% (unfavorable);
- Platelet change > 37% (unfavorable);
- Lactate change > 23% (unfavorable);
- Albumin change ≤ 7% (unfavorable).

This reflects the assumption that early non-survivors would have had the worst possible biomarker trajectories.

This approach provides a quantitative framework to illustrate the potential influence of survivorship bias on model performance and variable significance by defining realistic lower and upper bounds. When the results of all three scenarios are evaluated together, the “true” model performance is understood to lie between these two extremes. This structure also allows assessment of which variables are more or less sensitive to survivorship bias. Multicollinearity was assessed in all models, and model fit indices were reported.

## 3. Results

The in-hospital mortality rate among the 174 patients included in the study was 58.6%. The median age was 71 years (IQR: 56–82), and 59% of the patients were male. Comorbidities were present in 75.9% of the cohort, with a higher prevalence among non-survivors (82%). Among these, malignancy was significantly associated with increased mortality (*p* = 0.012). The demographic and clinical characteristics of survivors and non-survivors are summarized in Table 1.

Pneumonia was the most common source of infection (67.8%). No microbial growth was detected in 31% of cultures. Patients who received inappropriate empirical antibiotic therapy, defined as initial treatment not covering the isolated pathogen or not consistent with guideline-based empiric recommendations, had significantly higher in-hospital mortality rates (*p* = 0.001). Infection-related characteristics are summarized in Table 2.

Baseline severity scores differed between groups: the median SOFA score was higher among non-survivors [9 (IQR: 7–11)] compared with survivors [5 (IQR: 3–7)] (*p* < 0.001). Similarly, APACHE II scores were significantly higher in non-survivors [28 (IQR: 21–33) vs. 24 (IQR: 17–30), *p* = 0.040].

At 48 h, dynamic (Δ) changes in biomarkers were more pronounced among non-survivors compared with survivors. ΔProcalcitonin increased by a median of +44% in non-survivors, whereas it decreased by −17% in survivors (*p* < 0.001). ΔLactate increased by +8% in non-survivors but decreased by −7% in survivors (*p* = 0.008). ΔAlbumin decreased by −9% in non-survivors and by −5% in survivors (*p* = 0.015). The ΔCRP/albumin ratio increased by +30% in non-survivors compared with +8% in survivors (*p* = 0.049).

These results represent early biochemical trends within the first 48 h of ICU admission rather than immediate predictors, and they complement baseline severity scores (SOFA and APACHE II) rather than replace them. The detailed values are presented in Table 3.

Receiver operating characteristic (ROC) analysis was performed to assess the discriminative performance of 48 h biomarker changes (Δ) for in-hospital mortality prediction. The analysis identified the following optimal cutoff values: ΔProcalcitonin ≤ −28%, ΔLactate > +23%, ΔAlbumin ≤ −7%, and ΔCRP/Albumin > +31% (Table 4). Although these Δ-based AUC values did not exceed that of the baseline SOFA score, they provided complementary information that may assist in early risk stratification.

As shown in Table 4, the baseline SOFA score demonstrated the highest discriminative performance for in-hospital mortality (AUC = 0.755, 95% CI 0.684–0.817, *p* < 0.001). Among the 48 h biomarker changes, ΔProcalcitonin (AUC = 0.682), ΔLactate (AUC = 0.619), and ΔAlbumin (AUC = 0.608) showed moderate discriminatory ability. Although none of the biomarker-based AUC values surpassed that of the baseline SOFA score, these dynamic parameters provided complementary information reflecting the patient’s evolving clinical status. Based on the ROC-derived cutoff values, categorical stratification was performed to assess mortality risk (Table 5).

Univariate analysis revealed significant associations with in-hospital mortality for malignancy (*p* = 0.012), APACHE II score > 28 (*p* = 0.005), SOFA score > 6 (*p* < 0.001), inappropriate empirical antibiotic therapy (*p* = 0.001), ΔCRP > +26%, ΔProcalcitonin ≤ −28%, ΔLactate > +23%, ΔAlbumin ≤ −7%, ΔPlatelet ≤ −37%, and ΔCRP/albumin ratio > +31%.

### 3.1. Performance of the Primary Model and Independent Predictors

The multivariable logistic regression analysis of the primary cohort (174 patients who completed 48 h of follow-up) identified several strong independent predictors of mortality (Table 6). The model demonstrated excellent discriminatory capacity, with an AUC of 0.860 and an overall accuracy of 81.0%. The Nagelkerke R^2^ value (0.475) indicated that the model explained approximately 48% of the variance in the outcome. Model calibration was satisfactory, as confirmed by the goodness-of-fit statistics (Hosmer–Lemeshow *p* = 0.439).

Six prognostic variables that have been shown in the literature to be associated with mortality—SOFA score, 48 h change in procalcitonin, 48 h change in platelet count, 48 h change in lactate, 48 h change in albumin, and appropriateness of empirical antibiotic therapy—were included in the logistic regression analysis. Of the six examined variables, five were statistically significant: A SOFA score > 6 emerged as one of the strongest predictors (OR: 4.90; 95% CI: 2.21–10.85; *p* < 0.001), a >37% reduction in platelet count at 48 h was associated with the highest increase in mortality risk (OR: 6.52; 95% CI: 2.22–19.15; *p* < 0.001), a ≤28% decrease in procalcitonin at 48 h (OR: 3.78; 95% CI: 1.58–9.04; *p* = 0.003) and a >23% increase in lactate at 48 h (OR: 3.89; 95% CI: 1.58–9.54; *p* = 0.003) were identified as significant biomarker predictors. Inappropriate empirical antibiotic therapy, a measure of treatment quality, increased mortality risk by approximately fivefold (OR: 5.06; 95% CI: 1.42–18.10; *p* = 0.013). Change in albumin did not reach statistical significance (OR: 2.02; 95% CI: 0.93–4.40; *p* = 0.078) (Table 6).

### 3.2. Sensitivity Analyses for Survivorship Bias and Assessment of Model Robustness

The sensitivity analyses incorporating the 14 patients who died within the first 48 h—who therefore lacked 48 h biomarker change data—provided critical insight into the robustness of both the model and its individual predictors. These analyses were performed by imputing missing biomarker trajectories under a “best-case” assumption (Low-Risk Scenario, Supplementary Table S1) and a “worst-case” assumption (High-Risk Scenario, Supplementary Table S2).

### 3.3. Overall Model Performance

A comparative summary of performance metrics across all three scenarios is provided in Supplementary Table S3. The model’s discriminatory ability (AUC) decreased to 0.814 in the low-risk scenario but increased to 0.876 in the high-risk scenario, remaining above acceptable thresholds under all conditions. These findings indicate that the model’s overall discrimination is robust to survivorship bias.

### 3.4. Robustness of Individual Predictors

The most notable change at the variable level was observed for procalcitonin. While a strong predictor in the primary model (*p* = 0.003), it completely lost statistical significance in the low-risk scenario (*p* = 0.241). This suggests that the prognostic contribution of procalcitonin change is more susceptible to survivorship bias than the other biomarkers.

In contrast, SOFA score and antibiotic appropriateness retained high and stable odds ratios and remained significant under both sensitivity scenarios, demonstrating their robustness as predictors. Platelet and lactate changes remained statistically significant, although their effect sizes varied across scenarios.

### 3.5. Critical Changes in Performance Metrics

A more detailed comparison (Supplementary Table S3) demonstrated that survivorship bias does not affect all performance metrics equally. The most pronounced change was observed in specificity. In the low-risk scenario, specificity dropped sharply from 70.8% to 55.6%, suggesting that the model’s ability to correctly identify survivors may be substantially influenced by survivorship bias.

In contrast, sensitivity remained consistently high across all scenarios (88.2–89.7%), supporting the model’s reliability in identifying high-risk patients. Moreover, the Brier Score reached its lowest value (0.135) in the high-risk scenario, indicating optimal probability calibration and suggesting that the true clinical trajectory of these early non-survivors may align more closely with this assumption.

### 3.6. Additional Findings and Model Validity

As summarized in Supplementary Tables S4–S6, multicollinearity diagnostics demonstrated no evidence of collinearity among predictors, with tolerance values well above 0.9 and VIF values below 1.1, confirming the stability and reliability of the model coefficients.

### 3.7. Conclusion of Sensitivity Analyses

Overall, the developed logistic regression model demonstrated strong performance, with SOFA score, platelet change, and antibiotic appropriateness emerging as reliable and consistent predictors of sepsis mortality. However, the sensitivity analyses clearly indicated that the prognostic contribution of biomarker changes—particularly procalcitonin—and the model’s specificity may be influenced by survivorship bias. These findings underscore the importance of interpreting the model’s potential future clinical applications or validation efforts in light of these effects, especially with consideration of the performance patterns observed in the worst-case scenario.

## 4. Discussion

The prognostic assessment of sepsis requires not only baseline biomarker values but also their temporal changes, which may better capture the dynamic nature of the disease. Several prior studies have highlighted the prognostic significance of early biomarker kinetics, supporting the findings of our study. Jensen et al. reported that both elevated PCT levels and early increases within the first 24 h were independent predictors of 90-day mortality in the ICU, whereas CRP and leukocyte dynamics lacked prognostic value [20]. Similarly, Ruiz-Rodríguez et al. demonstrated that 48 h PCT clearance (PCTc) was the strongest with PCTc > 50%, associated with markedly improved outcomes [5]. Mat Nor et al. also showed that a PCTc greater than 30% at 48 h was independently associated with survival (HR = 2.90; 95% CI: 1.22–6.90) [21]. Consistent with these observations, our study showed that a decrease in PCT levels of less than 28% within the first 48 h was associated with increased mortality.

A similar trend has been reported for lactate levels. In the study by Li et al., changes in lactate patterns within the first 24 h were significantly associated with mortality, and patients who exhibited a decrease in lactate levels had higher survival rates. However, that study did not include data beyond the 24 h mark, such as 48 h measurements [22]. In a multicenter prospective cohort study conducted by Park et al., serial lactate monitoring demonstrated superior prognostic accuracy compared with the SOFA score alone. Notably, a decline in lactate levels between 48 and 72 h was significantly associated with improved survival, whereas an increase during the same interval was linked to an approximately fivefold higher risk of mortality [11]. In our study, a >23% increase in lactate during the first 48 h was independently associated with mortality. The relevance of lactate dynamics is further reinforced by findings from Karampela et al., who reported that nonsurvivors had persistently higher lactate levels both at sepsis onset and one week later, whereas lactate-to-albumin ratio (LAR) kinetics failed to distinguish survivors from nonsurvivors [23]. Likewise, Baldirà et al. showed that lactate had the highest predictive ability for 28-day mortality (AUROC 0.67), even in patients with SOFA ≤6 [24]. These results support the central prognostic role of lactate and align with the Δ-lactate cutoff determined in our analysis. Although the sensitivity and specificity derived from the ROC curves were moderate, the established thresholds still offer clinically meaningful insights that may aid in early risk stratification. However, these results should be interpreted as complementary to, rather than superior to, established scoring systems such as SOFA and APACHE II and, given the retrospective and single-center nature of the study, viewed as exploratory associations rather than validated predictors suitable for routine clinical decision-making.

Platelet dynamics have also been shown to play a significant role in the prognosis of sepsis. In a study by Si et al., temporal changes in platelet count were found to be closely associated with 60-day mortality, and persistent thrombocytopenia was identified as a potential indicator of poor prognosis [12]. Ye et al. similarly showed that serial platelet measurements were more informative than single-time assessments in predicting in-hospital mortality, with sustained platelet decline being strongly associated with an increased risk of death [25]. In our study, persistent thrombocytopenia and a lack of recovery in platelet counts within the first 48 h were independently associated with increased mortality, further supporting the prognostic utility of dynamic platelet monitoring in sepsis.

A key feature of our study is its evaluation of not only baseline biomarker levels but also early (48 h) dynamic changes in PCT, lactate, platelet count, and the CRP-to-albumin ratio in ICU patients. This time-based approach may enhance understanding of early physiological changes during sepsis. Still, these findings are associative and should not be interpreted as evidence that biomarker trends can reliably guide clinical decisions without further validation in larger, prospective cohorts.

This study has certain limitations. First, it was conducted in a single-center setting with a relatively small cohort, which may limit the external validity of the results. In addition, biomarker measurements were performed only up to 48 h, and longer-term dynamic changes were not evaluated. Furthermore, antibiotic appropriateness and timing may involve complex factors that could not be fully controlled.

Another important limitation is the potential selection bias arising from the exclusion of patients who died within the first 48 h of ICU admission. This group likely represents the most severely ill patients with rapid disease progression and refractory organ failure, whose pathophysiologic trajectory may differ from those who survived longer. The exclusion criterion, although methodologically necessary for Δ value calculations, may therefore limit the external validity of the findings. However, we addressed this limitation through structured sensitivity analyses incorporating early deaths under both best-case and worst-case assumptions, which demonstrated that while SOFA score, platelet change, and antibiotic appropriateness remained consistent and robust predictors, the prognostic contribution of PCT—along with model specificity—was influenced by survivorship bias. These sensitivity analyses were exploratory and were not pre-specified, but were performed to address reviewer-raised methodological concerns regarding survivorship bias. Additionally, differences in institutional sepsis recognition protocols and care-bundle implementation practices across centers may further contribute to variability in outcomes, thereby limiting generalizability.

Our study also demonstrated a relatively high overall mortality rate (58.6%), which exceeds the range reported in some previous sepsis cohorts. This finding likely reflects the characteristics of our study population, which consisted of elderly patients with a high prevalence of comorbidities, as well as the fact that the data were derived from a tertiary referral intensive care unit admitting severely ill cases. Rather than indicating a methodological weakness, this should be interpreted as a reflection of the local case mix and disease severity profile. Unlike centers that have implemented structured sepsis pathways, such as the PSC referenced in the Introduction, our institution does not employ a comparable system-level framework, which may partially explain the higher mortality observed in our cohort. Differences in institutional sepsis protocols and care-bundle implementation may therefore contribute to the variability in mortality rates across centers. Taken together, these factors underscore that our findings should be regarded as hypothesis-generating rather than definitive evidence of predictive performance. Future multicenter studies with larger sample sizes and prospective external validation will be essential to determine whether the associations identified here are reproducible and clinically applicable.

## 5. Conclusions

Our study demonstrated that 48 h changes in key biomarkers were independently associated with mortality in patients with sepsis. Multivariate analysis identified a SOFA score > 6, absence of appropriate empirical antibiotic therapy, a procalcitonin decrease ≤ 28%, a lactate increase > 23%, and a platelet count reduction > 37% as significant factors associated with increased mortality risk. While these biomarker dynamics do not replace established clinical scores, they may serve as complementary tools for early reassessment. However, given the retrospective single-center design and findings from sensitivity analyses—particularly the instability of PCT under survivorship bias—these associations should be interpreted cautiously and considered exploratory. If validated through prospective multicenter studies, the incorporation of 48 h biomarker changes into clinical protocols may enhance the precision of early risk assessment and support the development of individualized, data-driven treatment pathways in sepsis.

## Figures and Tables

**Table 1:** Comparison of demographic characteristics between survivors and non-survivors.

		Non-Survivors (*n* = 102)		Survivors (*n* = 72)				
		*n*	%	*n*	%	*p* ^¥^	OR (95%CI) ^ℒ^	*p*
Age (years) IQR		70 (60; 82)		72 (49; 83)				0.391
Sex	Female	48	47%	24	33%	0.070	1.78 (0.95–3.32)	0.071
	Male	54	53%	48	67%		1	
Comorbidity	Yes	84	82%	48	67%	**0.028**	**2.33 (1.15–4.73)**	**0.019**
	No	18	18%	24	33%		1	
Diabetes mellitus	Yes	27	26%	15	21%	0.499	1.37 (0.67–2.81)	0.393
	No	75	74%	57	79%		1	
Hypertension	Yes	47	46%	31	43%	0.693	1.13 (0.62–2.08)	0.693
	No	55	54%	41	57%		1	
Coronary artery disease	Yes	17	17%	8	11%	0.418	1.6 (0.65–3.94)	0.306
	No	85	83%	64	89%		1	
Congestive heart failure	Yes	7	7%	8	11%	0.478	0.59 (0.20–1.71)	0.330
	No	95	93%	64	89%		1	
Chronic kidney failure	Yes	11	11%	2	3%	0.092	4.23 (0.91–19.71)	0.066
	No	91	89%	70	97%		1	
Cerebrovascular event	Yes	5	5%	6	8%	0.549	0.57 (0.17–1.93)	0.365
	No	97	95%	66	92%		1	
Malignancy	Yes	24	24%	6	8%	**0.016**	**3.38 (1.31–8.78)**	**0.012**
	No	78	76%	66	92%		1	
COPD	Yes	12	12%	7	10%	0.858	1.24 (0.46–3.32)	0.671
	No	90	88%	65	90%		1	
Alzheimer	Yes	8	8%	4	6%	0.763	1.45 (0.42–5.00)	0.559
	No	94	92%	68	94%		1	
Other comorbidity	Yes	3	3%	2	3%	1.000	1.06 (0.17–6.51)	0.949
	No	99	97%	70	97%		1	

COPD: Chronic obstructive pulmonary disease, ^¥^ = Chi-Square Tests, ^ℒ^ = Mantel–Haenszel Common Odds Ratio Estimate. The bold values indicate statistically significant results (*p* < 0.05).

**Table 2:** Analysis of infection-related characteristics in survivors and non-survivors.

		Non-Survivors (*n* = 102)		Survivors (*n* = 72)				
		*n*	%	*n*	%	*p* ^¥^	OR (95%CI) ^ℒ^	*p*
Culture growth	Yes	68	67%	52	72%	0.539	0.77 (0.40–1.49)	0.436
	No	34	33%	20	28%		1	
Antibiotic appropriateness	Yes	75	74%	68	94%	**0.001**	1	**0.001**
	No	27	26%	4	6%		**6.12 (2.04–18.39)**	
Infection focus								
Pneumoniae	Yes	76	75%	47	65%	0.251	1.55 (0.80–3.00)	0.189
	No	26	25%	25	35%		1	
Bloodstream	Yes	16	16%	8	11%	0.523	1.49 (0.60–3.69)	0.391
	No	86	84%	64	89%		1	
Intra-abdominal	Yes	13	13%	11	15%	0.800	0.81 (0.34–1.93)	0.634
	No	89	87%	61	85%		1	
Genitourinary	Yes	3	3%	5	7%	0.382	0.41 (0.09–1.76)	0.228
	No	99	97%	67	93%		1	
Skin and soft tissue	Yes	1	1%	1	1%	n/a		
	No	101	99%	71	99%			

^¥^ = Chi-Square Tests, ^ℒ^ = Mantel–Haenszel Common Odds Ratio Estimate. The bold values indicate statistically significant results (*p* < 0.05).

**Table 3:** Comparison of 48 h changes (Δ) in clinical and laboratory parameters between survivors and non-survivors.

	Non-Survivors (*n* = 102)	Survivors (*n* = 72)	
	Median (IQR)	Median (IQR)	*p* *
APACHE II (Baseline)	28 (21; 33)	24 (17; 30)	**0.040**
SOFA (Baseline)	9 (7; 11)	5 (3; 7)	**<0.001**
ΔCRP	1.15 (0.85; 1.63) (+15% ↑)	0.99 (0.71; 1.35) (−1% ↓)	0.075
ΔProcalcitonin	1.44 (0.81; 3.13) (+44% ↑)	0.83 (0.49; 1.83) (−17% ↓)	**<0.001**
ΔLactate	1.08 (0.75; 1.60) (+8% ↑)	0.93 (0.71; 1.15) (−7% ↓)	**0.008**
ΔAlbumin	0.91 (0.84; 0.97) (−9% ↓)	0.95 (0.88; 1.03) (−5% ↓)	**0.015**
ΔNeutrophil	0.95 (0.69; 1.30) (−5% ↓)	0.88 (0.65; 1.15) (−12% ↓)	0.263
ΔLymphocyte	1.02 (0.74; 1.50) (+2% ↑)	0.90 (0.70; 1.38) (−10% ↓)	0.440
ΔPlatelet	0.79 (0.60; 1.10) (−21% ↓)	0.91 (0.74; 1.12) (−9% ↓)	0.066
ΔNLR	0.99 (0.59; 1.42) (−1% ↓)	0.97 (0.62; 1.40) (−3% ↓)	0.908
ΔPLR	0.83 (0.47; 133) (−17% ↓)	0.91 (0.69; 1.33) (−9% ↓)	0.156
ΔCRP/albumin	1.30 (0.91; 1.85) (+30% ↑)	1.08 (0.75; 1.48) (+8% ↑)	**0.049**

APACHE II: Acute Physiology and Chronic Health Evaluation II; SOFA: Sequential Organ Failure Assessment; CRP: C-reactive protein; NLR: Neutrophil-to-lymphocyte ratio; PLR: Platelet-to-lymphocyte ratio; Δ: Percentage change from baseline to 48 h post-admission for each parameter; IQR = Interquartile range; * Mann–Whitney Test; ↑ = Increase from baseline to 48th hour; ↓ = Decrease from baseline to 48th hour. The bold values indicate statistically significant results (*p* < 0.05).

**Table 4:** Receiver operating characteristic (ROC) analysis results for mortality prediction.

Variable	AUC (95% CI)	*p*_(Area = 0.5)_	J_Youden index_	Cut Off Value	Sensitivity %	Specificity %
APACHE II (Baseline)	0.592 (0.515–0.665)	0.038	0.20	>28	46	74
SOFA (Baseline)	0.755 (0.684–0.817)	<0.001	0.39	>6	76	63
ΔCRP (↑)	0.579 (0.502–0.654)	0.074	0.19	>26%	45	74
ΔProcalcitonin (↓)	0.682 (0.607–0.750)	<0.001	0.30	≤28%	84	46
ΔLactate (↑)	0.619 (0.542–0.691)	0.005	0.25	>23%	42	83
ΔAlbumin (↓)	0.608 (0.532–0.681)	0.013	0.23	>7%	64	60
ΔNeutrophil (↑)	0.55 (0.473–0.625)	0.262	0.11	>25%	29	82
ΔLymphocyte (↑)	0.534 (0.457–0.610)	0.439	0.14	≤9%	63	51
ΔPlatelet (↓)	0.582 (0.505–0.656)	0.056	0.23	>37%	32	90
ΔBilirubin (↑)	0.538 (0.461–0.614)	0.394	0.09	≥1%	46	63
ΔNLR (↑)	0.505 (0.428–0.582)	0.907	0.09	>53%	19	90
ΔPLR (↑)	0.563 (0.486–0.638)	0.146	0.22	>44%	41	81
ΔCRP/Albumin (↑)	0.588 (0.511–0.662)	0.047	0.19	>31%	50	69

APACHE II: Acute Physiology and Chronic Health Evaluation II; SOFA: Sequential Organ Failure Assessment; CRP: C-reactive protein; NLR: Neutrophil-to-lymphocyte ratio; PLR: Platelet-to-lymphocyte ratio; Δ: Percentage change from baseline to 48 h post-admission for each parameter; ROC: Receiver Operating Characteristic; AUC: Area Under the Curve; ↑ = Increase from baseline to 48th hour; ↓ = Decrease from baseline to 48th hour.

**Table 5:** Mortality analysis according to ROC-derived cutoff values.

	Non-Survivors (*n* = 102)		Survivors (*n* = 72)				
	*n*	%	*n*	%	*p* ^¥^	OR (95%CI) ^ℒ^	*p*
APACHE II (Baseline > 28)	50	49%	20	28%	**0.005**	**2.50 (1.31–4.77)**	**0.005**
SOFA (Baseline > 6)	78	76%	27	38%	**<0.001**	**5.42 (2.80–10.49)**	**<0.001**
ΔCRP (↑ > +26%)	46	45%	19	26%	**0.012**	**2.29 (1.19–4.40)**	**0.013**
ΔProcalcitonin (↓ ≤ −28%)	86	84%	39	54%	**<0.001**	**4.55 (2.24–9.22)**	**<0.001**
ΔLactate (↑ > +23%)	43	42%	12	17%	**0.001**	**3.64 (1.75–7.59)**	**0.001**
ΔAlbumin (↓ ≤ −7%)	65	64%	29	40%	**0.002**	**2.60 (1.40–4.84)**	**0.002**
ΔPlatelet (↓ ≤ −37%)	32	31%	7	10%	**0.001**	**4.24 (1.75–10.28)**	**0.001**
ΔCRP/Albumin (↑ > +31%)	51	50%	22	31%	**0.010**	**2.27 (1.21–4.28)**	**0.011**

APACHE II: Acute Physiology and Chronic Health Evaluation II; SOFA: Sequential Organ Failure Assessment; CRP: C-reactive protein; NLR: Neutrophil-to-lymphocyte ratio; PLR: Platelet-to-lymphocyte ratio; Δ: Percentage change from baseline to 48 h post-admission for each parameter; ^¥^ = Chi-Square Tests; ^ℒ^ = Mantel–Haenszel Common Odds Ratio Estimate; ↑ = Increase from baseline to 48th hour; ↓ = Decrease from baseline to 48th hour. The bold values indicate statistically significant results (*p* < 0.05).

**Table 6:** Primary Model Logistic Regression Analysis (48 h Cohort, *n* = 174).

Variable	β	SE	*p*-Value	OR	95% CI
SOFA > 6	1.590	0.405	<0.001	4.90	2.21–10.85
Procalcitonin ↓% ≤ 28	1.330	0.445	0.003	3.78	1.58–9.04
Platelet ↓% > 37	1.875	0.550	<0.001	6.52	2.22–19.15
Lactate ↑% > 23	1.358	0.458	0.003	3.89	1.58–9.54
Albumin ↓% > 7	0.702	0.398	0.078	2.02	0.93–4.40
Inappropriate Antibiotic	1.622	0.650	0.013	5.06	1.42–18.10
Constant	−2.835	0.541	<0.001	0.06	0.02–0.17

Model Performance: Nagelkerke R^2^ = 0.475, AUC = 0.860, Accuracy = 81.0%. Model Fit: Omnibus χ^2^ = 75.746, *p* < 0.001; Hosmer–Lemeshow χ^2^ = 7.948, p = 0.439. SOFA: Sequential Organ Failure Assessment.

## Data Availability

The data supporting this study’s findings are available on request from the corresponding author. The data are not publicly available due to privacy or ethical restrictions.

## Supplementary Materials

The following supporting information can be downloaded at: https://www.mdpi.com/article/10.3390/jcm14248651/s1, Table S1: Low-Risk Scenario Logistic Regression Analysis (*n* = 188); Table S2: High-Risk Scenario Logistic Regression Analysis (*n* = 188); Table S3: Performance Comparison Across Sensitivity Analysis Scenarios; Table S4: Multicollinearity Analysis Across All Regression Models; Table S5: Multicollinearity Assessment Criteria; Table S6: Detailed Performance Metrics Comparison Across All Models.
