# Supplementary material for: Prognostic Value of 48-h Biomarker Changes in Sepsis Mortality
Source: J Clin Med. 2025 Dec 6;14(24):8651. doi: 10.3390/jcm14248651 (PMC12733996; doi:10.3390/jcm14248651)
Supplement: Supplementary file 1 [file jcm-14-08651-s001.zip › jcm-4008738-supplementary.pdf]

Supplementary Table S1: Low-Risk Scenario Logistic Regression Analysis (*n*=188)

Variable	β	SE	p-value	OR	95% CI
SOFA >6	1.709	0.366	<0.001	5.53	2.70 - 11.33
Procalcitonin ↓% ≤28	0.441	0.376	0.241	1.55	0.74 - 3.25
Platelet ↓% >37	1.300	0.497	0.009	3.67	1.39 - 9.73
Lactate ↑% >23	1.098	0.437	0.012	3.00	1.27 - 7.06
Albumin ↑% >7	0.126	0.364	0.729	1.13	0.56 - 2.32
Inappropriate Antibiotic	1.627	0.614	0.008	5.09	1.53 - 16.94
Constant	-1.583	0.397	<0.001	0.21	0.09 - 0.45

SOFA: Sequential Organ Failure Assessment
Model Performance: Nagelkerke $R^2 = 0.363$*, AUC = 0.814, Accuracy = 76.6%*
Model Fit: Omnibus $\chi^2 = 58.471$*,* $p < 0.001$*; Hosmer-Lemeshow* $\chi^2 = 13.508$*,* $p = 0.096$
The Low-Risk Scenario included the 14 patients who died before the 48-hour time point. For these patients, missing 48-hour biomarker change values were classified into the favorable category (i.e., the category associated with better outcomes: Procalcitonin decrease >28%, Platelet decrease ≤37%, Lactate increase ≤23%, Albumin increase >7%). This represents an optimistic assumption about the potential biomarker trends of early deceased patients.

Supplementary Table S2: High-Risk Scenario Logistic Regression Analysis (*n*=188)

Variable	β	SE	p-value	OR	95% CI
SOFA >6	1.589	0.406	<0.001	4.90	2.21 - 10.86
Procalcitonin ↓% ≤28	1.368	0.445	0.002	3.93	1.64 - 9.40
Platelet ↓% >37	1.982	0.536	<0.001	7.26	2.54 - 20.75
Lactate ↑% >23	1.418	0.454	0.002	4.13	1.70 - 10.05
Albumin ↑% >7	0.742	0.397	0.061	2.10	0.97 - 4.57
Inappropriate Antibiotic	1.613	0.653	0.013	5.02	1.40 - 18.02
Constant	-2.900	0.539	<0.001	0.06	0.02 - 0.16

SOFA: Sequential Organ Failure Assessment
Model Performance: Nagelkerke $R^2 = 0.513$*, AUC = 0.876, Accuracy = 82.4%*
Model Fit: Omnibus $\chi^2 = 89.190$*,* $p < 0.001$*; Hosmer-Lemeshow* $\chi^2 = 9.214$*,* $p = 0.325$
The High-Risk Scenario included the 14 patients who died before the 48-hour time point. For these patients, missing 48-hour biomarker change values were classified into the unfavorable category (i.e., the category associated with worse outcomes: Procalcitonin decrease ≤28%, Platelet decrease >37%, Lactate increase >23%, Albumin increase ≤7%). This represents a conservative assumption about the potential biomarker trends of early deceased patients.

Supplementary Table S3: Performance Comparison Across Sensitivity Analysis Scenarios

Performance Metric	Primary Model	Low-Risk Scenario	High-Risk Scenario
Sample Size (*n*)	174	188	188
Nagelkerke R^2	0.475	0.363	0.513
AUC	0.860	0.814	0.876
Overall Accuracy	81.0%	76.6%	82.4%
Sensitivity	88.2%	89.7%	89.7%
Specificity	70.8%	55.6%	70.8%
Omnibus Test (p)	<0.001	<0.001	<0.001
H-L Test (p)	0.439	0.096	0.325

Note: The low-risk scenario assumed favorable biomarker changes for early deceased patients; the high-risk scenario assumed unfavorable changes. All models showed excellent fit (Hosmer-Lemeshow $p > 0.05$).

Supplementary Table S4: Multicollinearity Analysis Across All Regression Models

Variable	Primary Model (*n*=174)	Low-Risk Scenario (*n*=188)	High-Risk Scenario (*n*=188)
	Tolerance / VIF	Tolerance / VIF	Tolerance / VIF
SOFA >6	0.950 / 1.053	0.953 / 1.049	0.948 / 1.055
Procalcitonin ↓% ≤28	0.936 / 1.068	0.943 / 1.060	0.944 / 1.060
Platelet ↓% >37	0.916 / 1.091	0.992 / 1.008	0.937 / 1.067
Lactate ↑% >23	0.950 / 1.053	0.920 / 1.087	0.954 / 1.049
Albumin ↑% >7	0.963 / 1.038	0.954 / 1.048	0.973 / 1.027
Inappropriate Antibiotic	0.967 / 1.035	0.969 / 1.032	0.966 / 1.035

SOFA: Sequential Organ Failure Assessment

Interpretation: All Variance Inflation Factor (VIF) values were well below the conservative threshold of 5 (and even 2), and Tolerance values were all well above 0.1, indicating no concerning multicollinearity in any of the tested models.

Supplementary Table S5: Multicollinearity Assessment Criteria

Criterion	Current Study Range	Interpretation
Tolerance	0.916 - 0.992	Excellent
VIF	1.008 - 1.091	Excellent
Assessment	-	No multicollinearity

Supplementary Table S6: Detailed Performance Metrics Comparison Across All Models

Performance Metric	Primary Model (*n*=174)	Low-Risk Scenario (*n*=188)	High-Risk Scenario (*n*=188)
**Model Fit Statistics**			
Omnibus Test	$\chi^2 = 75.75$, $p < 0.001$	$\chi^2 = 58.47$, $p < 0.001$	$\chi^2 = 89.19$, $p < 0.001$
Hosmer-Lemeshow Test	$\chi^2 = 7.95$, $p = 0.439$	$\chi^2 = 13.51$, $p = 0.096$	$\chi^2 = 9.21$, $p = 0.325$
**Explanatory Power**			
Nagelkerke R^2	0.475	0.363	0.513
**Discrimination & Accuracy**			
AUC	0.860	0.814	0.876
Overall Accuracy	81.0%	76.6%	82.4%
Sensitivity	88.2%	89.7%	89.7%
Specificity	70.8%	55.6%	70.8%
**Calibration**			
Brier Score	0.146	0.170	0.135

Interpretation: All models demonstrated excellent overall fit (Omnibus $p < 0.001$) and good calibration (Hosmer-Lemeshow $p > 0.05$). The primary model showed excellent discrimination (AUC > 0.85) and calibration (Brier Score = 0.146), which remained robust across sensitivity analysis scenarios. The Brier Score was lowest in the high-risk scenario (0.135) and highest in the low-risk scenario (0.170), indicating better probability calibration when assuming unfavorable biomarker trends for early deceased patients.
